# A retrospective analysis and literature review of neoplastic appendiceal mucinous lesions

**DOI:** 10.1186/s12893-021-01091-9

**Published:** 2021-02-11

**Authors:** Belén Matias-García, Fernando Mendoza-Moreno, Ana Blasco-Martínez, José Ignacio Busteros-Moraza, Manuel Diez-Alonso, Francisca Garcia-Moreno Nisa

**Affiliations:** 1 Surgery Department, Hospital Universitario Príncipe de Asturias, Alcalá de Henares, 28005 Spain; 2 Pathology Department, Hospital Universitario Príncipe de Asturias, Alcalá de Henares, 28805 Spain; 3 Surgery and Medical Sciences Deparment, GIBIT-UAH CIBER-BBN, Alcala University, Campus Universitario, 28805 Alcalá de Henares, Madrid Spain

**Keywords:** Appendix, Mucinous neoplasm, Low-grade appendiceal mucinous neoplasm, Mucocele

## Abstract

**Background:**

At present, the term mucocele is outdated, and mucinous appendiceal neoplasm is preferred. Mucinous appendiceal neoplasm is an uncommon pathology that occurs predominantly in middle-aged women. Its classification and management have been the subject of debate in recent decades. The aim of this study was to analyse the incidence, clinical management and survival of these tumours diagnosed in our centre in the last 10 years.

**Methods:**

This was a retrospective observational study of patients with a diagnosis of appendiceal neoplasms between 2009 and 2018 in our centre. Variables such as sex, age, tumour type, clinical status, diagnosis, treatment and survival were collected. All data were analysed using the statistical program IBM SPSS Statistic® version 25.

**Results:**

Twenty-nine patients with a diagnosis of appendiceal neoplasm were identified, and 24 corresponded to neoplastic appendiceal mucinous lesions (85.7%). The average age was 59.7 ± 17.6 years. Most patients were women (15 cases; 62.5%). Most of them presented with chronic abdominal pain (37.5%), and the diagnosis was performed by computed tomography (CT) (50%). The treatment was surgical in all cases. The surgical technique depended on the findings and histology of the tumour.

**Conclusion:**

Mucinous appendiceal neoplasms are an uncommon entity, and their pathological classification and management have recently changed.

## Background

The term mucocele refers to a cystic dilation of the appendix with accumulation of mucinous material [1, 2]. It may be caused by a benign or malignant process [1–3]. Currently, the term mucocele is outdated, and the term neoplastic appendiceal mucinous lesions is preferred. These lesions are an uncommon disease. The most frequent form of presentation is pain in the right iliac fossa, mimicking acute appendicitis. Therefore, the definitive diagnosis is obtained from the pathological exam. However, it is not uncommon to find asymptomatic patients who are diagnosed incidentally in the course of another examination [4, 5]. In advanced cases, the disease can spread to the peritoneal cavity in the form of semisolid adhesive mucin, making the tumor prognosis much worse.

Currently, the pathological classification and nomenclature of the condition continue to be the subject of debate, implying a lack of consensus on both treatment and follow-up in daily medical practice in these patients. The latest classification of the World Health Organization 2019 (WHO) divides them into. Serrated polyps, hyperplatic polyps, low-grade appendiceal mucinous neoplasms (LAMNs), high-grade appendiceal mucinous neoplasmas (HAMs), and mucinous adenocarcinomas [6]. To this classification, the eighth edition of the American Committee on Cancer (AJCC 8^th^ Ed.) adds the concept of high-grade appendiceal mucinous neoplasm (HAMN) for lesions without infiltrative invasion but with high-grade cytologic atypia [5].

Recently, the consensus established by the members of the Peritoneal Surface Oncology Group International (PSOGI) Executive Committee in 2020 divided epithelial mucinous neoplasms of the appendix into serrated polyps, LAMN, HAMN, and mucinous adenocarcinoma (with or without signet ring cells) [7].

The aim of this study was to analyse these tumors diagnosed in our centre in the last 10 years.

## Methods

This was a retrospective observational study of patients with a diagnosis of appendiceal neoplasms between 2009 and 2018 in our centre. Cases in which the pathological anatomy (PA) was reported as appendiceal tumor or neoplasm were selected. Patients older than 18 years with a diagnosis of epithelial mucinous neoplasia were selected. Exclusion criteria included patients with a previous diagnosis of inflammatory bowel disease, nonmucinous epithelial neoplasms, epithelial neoplasms with neuroendocrine features, mesenchymal appendicular neoplasms and inflammatory tumors. Pathological anatomy preparations were reviewed and classified based on the WHO classification.

Data were tabulated in a computerized database (IBM SPSS Statistic® version 25). In the case of categorical variables, the proportion of each category with respect to the total number of patients was calculated. For qualitative variables, the distribution of phenomena was studied. For quantitative variables, the average and standard deviation were studied.

Variables such as sex, age, type of neoplasm, clinical manifestations, tumor markers (positivity and evolution in the follow-up), diagnosis (CT scan, colonoscopy, exploratory laparoscopy and pathological anatomy), treatment (urgent or elective) and surgical intervention, cytology, reintervention and type of surgery, presence of other colorectal neoplasm, presence of other neoplasm, deaths 30 days after the intervention and survival at 5 years were analysed. The follow-up in these patients was carried out using tumor markers and CT.

## Results

A total of 154 patients with a diagnosis of appendiceal neoplasm were identified; 125 were inflammatory processes (appendicitis, retention cysts or hyperplasia appendicular). Of the 29 patients in our series: 20 were low-grade mucinous neoplasms (83.3%), 1 high-grade mucinous neoplasm (4.2%) and 3 mucinous adenocarcinomas (12.5%). Of these patients, 1 LAMN and 1 HAMN presented with PMP at diagnosis (1 high-grade and 1 low-grade mucinous carcinoma peritoneum). The other 5 cases were tumors that did not meet criteria and inclusion in the study.

Of the 24 patients with mucinous neoplasms, 15 were women (62.5%). The average age was 59.7 years (median age 57 years). The main symptom was chronic abdominal pain in 9 patients (37.5%); 7 patients presented acute abdominal pain in the right iliac fossa mimicking appendicitis, and 1 patient presented with digestive bleeding. The 7 remaining patients were asymptomatic and were diagnosed as incidental findings in an imaging test.

The diagnosis was mainly performed by CT scan (50% of patients). In 37.5%, diagnosis was obtained by pathological anatomy. The most frequent finding in the CT scan was a dilation of the appendix (53.8%), followed by 46.1% appendicular tumors and 30.7% calcifications (several patients presented more than one finding). Only two patients were diagnosed in the context of exploratory laparoscopy, and only one patient was diagnosed by colonoscopy, showing an ulcerated appendicular mucosa.

Tumor markers were studied in 17 patients. CEA, CA19.9 and CA125 were elevated in only one case. The evolution of these markers could not be followed due to the death of the patient.

The treatment was surgical in all cases, with elective surgery in 16 patients (66.7%) and emergency surgery in 8 patients (33.3%) due to acute abdomen (Table 1).

**Table 1:** Surgical treatment of mucocele in urgent and elective surgery

	Urgent n (%)	Elective n (%)
Open appendectomy	5 (62.5)	5 (31.3)
Laparoscopic appendectomy	1 (12.5)	4 (25)
Open right hemicolectomy	1 (12.5)	3 (18.8)
Laparoscopic right hemicolectomy	0 (0)	2 (12.5)
Cytoreduction and HIPEC	0 (0)	2 (12.5)
Subtotal colectomy	1 (12.5)	0 (0)
Total	8 (33.3)	16 (66.7)

Concerning patients who underwent emergency surgery, six appendectomies were performed in 6 patients, with 5 open surgeries (62.5%) and 1 laparoscopic surgery (12.5%). All of them were LAMN, and the last one had to be reoperated due to the involvement of margins in the surgical piece. A right laparoscopic hemicolectomy was performed. This patient is currently disease-free. Of the 2 remaining patients who underwent emergency surgery, one was treated by open right colectomy due to involvement of the appendicular base (12.5%). In the other patient, a subtotal colectomy (12.5%) was performed due to sepsis secondary to pseudomembranous colitis. In both patients, a mucinous adenocarcinoma was found in the surgical piece.

Among the 16 patients who underwent elective surgery, appendectomies were performed in 9 of them (5 open (31.3%) and 4 laparoscopic (25%)). All of them were LAMN. One patient was diagnosed with diffuse peritoneal disease in an exploratory laparoscopy performed by the Gynaecology Department due to suspected ovarian neoplasm. A laparoscopic appendectomy was performed during the same surgical procedure. Subsequently, she had to be intervened to complete cytoreduction and treatment with HIPEC (Hipertermic Peritoneal Chemotherapy). This patient has presented a survival of 43 months and is currently free of disease.

Right colectomy was performed in 5 patients (2 laparoscopic (12.5%) and 3 open (18.8%)). Four of them were LAMN and one was mucinous adenocarcinoma. In one of the patients in whom an open right colectomy was performed, treatment was completed with cytoreduction + HIPEC due to mucin exiting into the peritoneal cavity during surgery.

In another 2 patients with an initial diagnosis of diffuse peritoneal disease (12.5%), right colectomy, central peritonectomy, omentectomy, hysterectomy and double anexectomy (in the case of a female patient), and HIPEC with mitomycin C for 90 min at 42 °C were performed. One of them was a LAMN, whereas the other was a HAMN. The last patient died in the immediate postoperative period due to pulmonary thromboembolism.

Postoperative complications according to the Clavien–Dindo classification were one grade I (prolonged paralytic ileus) and two grade V complications. One of them died in the immediate postoperative period due to pulmonary thromboembolism, as previously mentioned. Another patient died from unknown causes without evidence of recurrence until that moment.

The median follow-up of these patients was 15 months. No recurrence was evident in any patient. No patient received postoperative chemotherapeutic treatment.

Regarding the presence of other digestive neoplasms, only one patient with mucinous neoplasm also presented a synchronous right colon neoplasm and was treated by right colectomy. In relation to other non-digestive neoplasms, 12.5% of our patients had presented another neoplasm prior to the diagnosis of appendiceal neoplasm. One patient presented an ovarian neoplasm, and another 2 patients presented with breast cancer.

## Discussion

Appendiceal mucinous lesions are classified into two major groups of non-neoplastic lesions (mucocele) and neoplastic lesions (serrated polyps, hyperplastic polyps, LAMN, HAMN, and mucinous adenocarcinomas). Neoplastic appendiceal mucinous lesions are an uncommon pathology seen in 0.2–1.4% of appendectomies. It is usually diagnosed between 50 and 60 years [1–5, 9, 10]. The term mucocele refers to cystic dilation of the appendix with accumulation of mucinous material that may be due to a proximal obstruction (a coprolite or tumor) or may be caused by mucus-secreting neoplasms [1–3, 10]. Appendiceal mucocele was first described in 1842 by Rokitansky and now the term mucocele is outdated. The classification of appendiceal mucinous neoplasm constitutes a very broad diagnosis ranging from adenoma to mucinous adenocarcinoma. For this reason, these has been a need for the classifications regarding appendiceal mucinous neoplasms to be re-examined [11]. Since simple mucocele/simple retention cyst/inflammatory mucocele, or obstructive mucocele do not refer to a neoplastic lesion, it does not exist in WHO classification of tumors of the digestive system 2019. [8]

**Fig. 1:**
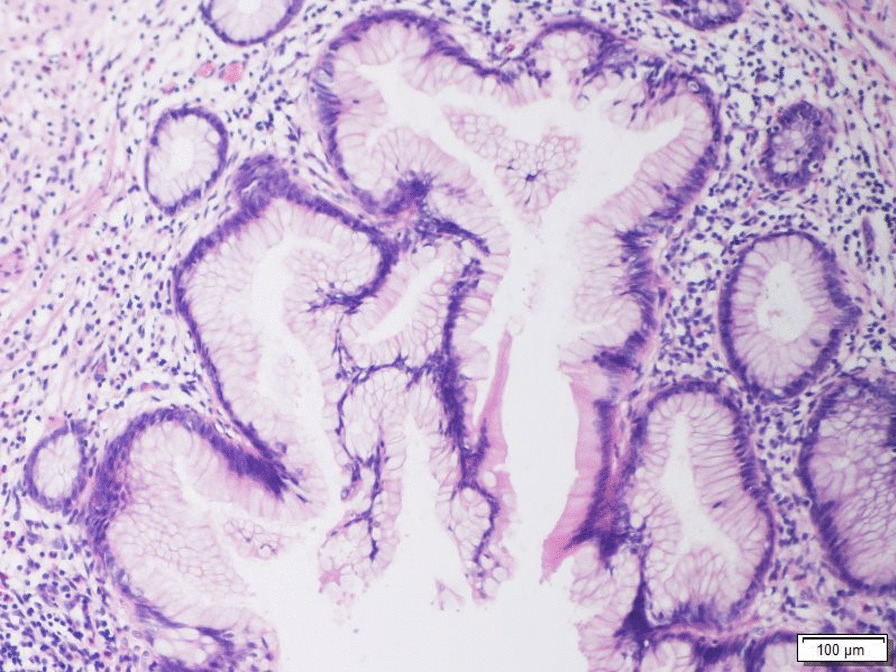
Histological section of a low-grade appendiceal mucinous neoplasm

From the pathological point of view, the 2019 WHO classification divides these tumors into LAMN, HAMN and mucinous adenocarcinomas. LAMNs do not present infiltrative epithelial invasion of the appendiceal wall and are limited to the muscularis propia. Appendiceal tumors such as LAMNs, HAMNs, and mucinous adenocarcinomas can lead to peritoneal spread. It can happen after the rupture of the appendix, the clinical entity called pseudomixoma peritonei (PMP) appears [6] (Fig. 1).

To this classification, the eighth edition of the AJCC adds the concept of HAMN. Both LAMN and HAMN lack infiltrative invasion, but the latter presents high-grade cytologic atypia [6, 10, 12]. However, the dilemma about the classification and nomenclature of this pathology continues to be the subject of debate and consequently generates controversy for the most appropriate management in daily clinical practice. Prognostic parameters include the presence of symptoms, perforation, operation type, increased tumor marker levels, and presence of a tumor at surgical borders for appendiceal mucinous neoplasm [12, 13]. Bell et al. conclude that LAMNs are bland and indolent neoplasms when confined to the appendix, without complicating factors such rupture or PMP. They present a 117 cases series and only 1 of 117 cases led to pseodomixoma [14].

In February 2020, PSOGI members established a consensus regarding the classification and treatment of these malignancies. This classification includes LAMN, HAMN, and mucinous adenocarcinoma and considers serrated polyps as appendicular mucinous neoplasms [15].

PMP is a peritoneal accumulation of mucinous substance secondary to the rupture of an appendicular lesion of this type [5, 6, 10]. The latest PSOGI classification divides PMP into 4 subtypes [15]: acellular mucin (M1a in 8th ed AJCC) (mucin without neoplastic epithelium), low-grade mucinous carcinoma peritonei (M1b G1 in 8th ed AJCC) (cytologically low grade, few mitoses and mucinous epithelium is scant (< 20% tumor volume)), high-grade mucinous carcinoma peritonei (M1b G2 or G3 in 8th ed AJCC) (abundant neoplastic mucinous epithelium (> 20% tumor volume) and the presence of one or more of the following: high-grade cytology, infiltrative invasion into adjacent tissue, angiolymphatic or perineural invasion, cribriform growth), and high-grade mucinous carcinoma peritonei with signet ring cells (M1b G3 in 8th ed AJCC).

Patients are often asymptomatic or have nonspecific symptoms, and the diagnosis is made incidentally in the course of another examination [4, 5]. However, the most frequent clinical forms of presentation are pain in the right iliac fossa mimicking acute appendicitis (more than 50% of cases), abdominal mass in the right iliac fossa, gastrointestinal bleeding or intestinal obstruction [1, 2, 4, 5, 16, 17]. In our series, most of the patients presented chronic abdominal pain (37.5%, n = 9), followed closely by the group of patients who presented acute abdominal pain in the right iliac fossa (29.2%, n = 7) and the group of patients who were asymptomatic at diagnosis (29.2%, n = 7). However, although the appendix appears to look normal, histopathological assessment of specimens is required to rule out malignant and infectious appendiceal diseases [17]. Yilmaz et al. reports that 8.3% of the patients who received appendicectomy to treat the initial diagnosis of acute appendicitis had unusual histopathological findings in their appendectomy specimens [18]. In women diagnosed with primary mucinous ovarian tumor with bilateral ovarian lesion, differential diagnosis with appendicular neoplastic mucinous lesions should be considered, especially if the endoscopic digestive study is normal [19].

For diagnosis, the measurement of tumor markers (CEA, CA19.9, CA125) may be useful. There are series in which up to 67% of patients may have elevated markers [4, 5]. Although there is a paucity of information, the available data suggest that tumor markers are elevated in the majority of patients with advanced appendiceal mucinous tumors, ad the levels correlate with treatment outcomes [20]. In addition, these markers are useful in terms of prognosis because elevated levels at the time of appendectomy could indicate an increased risk of recurrence or death [21]. The latest PSOGI recommendations regarding PMP promote the baseline determination of CEA and CA 19.9 in serum on a mandatory basis, while CA 125 could also be evaluated [15]. Only one patient presented elevated tumor markers at diagnosis, including CEA, CA19.9 and CA125. The evolution could not be followed due to the patient's death in the immediate postoperative period. In our sample, follow-up was performed with physical examination and CT scan.

**Fig. 2:**
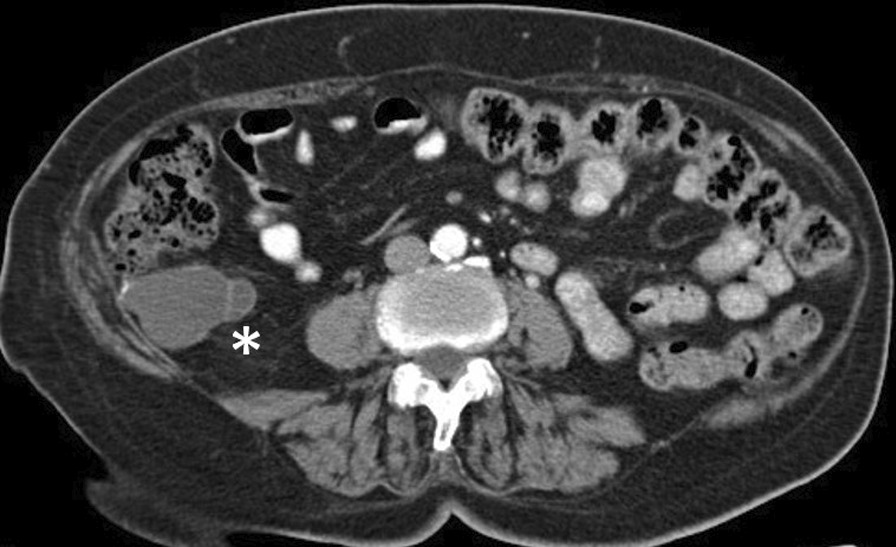
CT scan showing low-grade appendiceal mucinous neoplasms with liquid density adjacent to the caecum and retrocecum (*)

However, the gold standard preoperative diagnostic imaging test is CT scan [4, 15, 16], which shows a cystic mass of liquid density adjacent to the caecum and a retrocecal location in most cases [4, 16] (Fig. 2).

Visualization of the appendix and an increase of > 15 mm in its size suggested specific appendiceal mucocele with 83% sensitivity and 92% specificity. Preoperative imaging studies were performed in eight of our cases, and these had similar findings. The major criterion for discrimination between appendicitis and appendiceal mucinous neoplasm is a wall thickness > 6 mm. The clinical signs of appendicitis and the clinical picture of mucin leakage following mucinous neoplasm rupture often cannot be distinguished from each other [11]. The finding of calcifications in the wall is very suggestive of mucinous neoplasia but is found in less than 50% of cases [3–5, 10]. Of the total of 24 patients, 12 were intervened according to the CT findings (50%). The most common finding was dilation of the appendix (53.8%), followed closely by mass effect/appendicular tumor (46.1%) and calcifications (30.7%). The greater the diameter of the lesion as observed on CT scan, the more accurate the diagnosis [22]. In addition, CT scans also allow us to evaluate the extent of the disease and to diagnose complications, including inflammation, invagination, torsion, compression of the ureter and the presence of peritoneal disease [1, 3, 10]. Since appendiceal mucinous neoplasms are often diagnosed after the age of 50 years, CT studies are recommended for patients over this age presenting with signs of appendicitis [11].

In the case of peritoneal disease, magnetic resonance imaging and PETCT may also be useful as diagnostic imaging tests in the preoperative evaluation. Laparoscopic resecability evaluation in the preoperative study of patients was expected to include peritoneal disease [15].

The treatment of neoplastic appendiceal mucinous lesions is surgical for two reasons: its possible malignancy and the possibility of rupture in 5 to 15% of cases, with risk of dissemination and progression to pseudomyxoma peritonei [3]. The surgical procedure must be related to the findings of the tumor (extension, presence of mucus in the peritoneum, rupture of the appendix or safety margins) and its histology [1]. There is no standard treatment protocol for cases with surgical border positivity [11].

In case of an unexpected finding of PMP during the course of elective abdominal surgery, the surgeon should abort the procedure and take biopsies for the histological diagnosis of peritoneal disease and primary appendiceal neoplasm. The treatment of PMP will be different depending on each subtype [15]. Even if LAMNs are spread across the peritoneum, the lymph nodes are generally not affected. Therefore, the role of right hemicolectomy in this patient with widespread peritoneal disease is unclear [23] Appendicectomy alone is curative for benign and grossly intact mucinous neoplasm. Right hemicolectomy is only recommended when there is a risk of ileocecal valve injury due to traumatic manipulation or protrusion of the tumor toward the cecal lumen [11].

According to the latest PSOGI recommendations, oxaliplatin could be used for HIPEC instead of mitomycin C. The use of neoadjuvant chemotherapy in PMP should be considered in patients with high-grade PMP or PMP with signet ring cells. The ideal regimen is the combination of fluoropyrimidin and alkylating agents such as oxaliplatin. The use of adjuvant chemotherapy should be considered in patients with high-grade PMP or seal ring cell PMP in whom complete cytoreduction (R0-1) and HIPEC have been performed. The adjuvant chemotherapy regimen would be the same as in neoadjuvant therapy [15].

In all our patients undergoing HIPEC, the regimen used was mitomycin C for 90 min at 42 °C. Folate and 5-FU were previously administered intravenously.

For the follow-up of patients undergoing cytoreduction and HIPEC, recommendations include physical examination and thoracic-abdominal-pelvic CT scan every 6 months during the first 2 years. Subsequently, they recommend a physical exam every 6 months and thoracic-abdominal-pelvic CT annually. The use of tumour markers during follow-up is recommended at a frequency of 6 months [15].

The role of laparoscopy in these tumors is controversial. Low-grade neoplasms require careful and a traumatic management during extraction to minimize the risk of rupture and peritoneal seeding [5, 10]. There are authors who defend laparotomy as the surgical approach of choice for these reasons, indicating conversion to laparotomy in the presence of an appendicular neoplasm during laparoscopy. [1–3, 10]. However, laparoscopic approaches have been used successfully for resection of mucinous neoplasms, and prolonged follow-up has shown no increase in recurrence [1, 5]. Therefore, laparoscopic surgery may be of choice in selected patients, without evidence of rupture and dissemination [3, 4, 10].

Of the 5 patients who underwent laparoscopic appendectomy, two had to be reoperated. One of them was operated on in an emergent context due to symptoms suggestive of appendicitis and had to be reoperated due to compromise of the surgical margin. A right laparoscopic hemicolectomy was performed. The other patient was diagnosed with pseudomyxoma peritonei in an exploratory laparoscopy performed by the Gynaecology Department due to suspected ovarian neoplasm. Laparoscopic appendectomy was performed during the same surgical procedure, and she underwent surgery to complete cytoreduction and HIPEC. These two patients are currently disease-free. In patients in whom possible margin involvement was suspected, a right colectomy was performed as an initial treatment. The margins of the surgical piece were clear, and all patients are currently free of disease.

The prognosis depends on whether there is progression to advanced forms. In benign forms, progression to peritoneal dissemination only occurs in approximately 2%, while up to 23% of mucinous adenocarcinomas progress to this entity. Without this progression, the 5-year survival of the benign forms is almost 100%, while that of the malignant forms varies from 30 to 80% [3]. However, the 5-year survival for PMP varies from 23 to 77% in reference centres [5, 7]. According to Fish et al. [7], the overall survival rates at 5 and 10 years of patients undergoing cytoreduction and HIPEC due to disseminated disease were 77% and 66%, respectively. Incomplete cytoreduction was associated with significantly lower survival compared to complete cytoreduction. In our series, we cannot draw significant conclusions due to the insufficient sample size.

These types of tumors are associated with 20% of colorectal neoplasms; therefore, screening is recommended by performing a colonoscopy [10]. In our sample, only one patient presented with associated colorectal cancer (4.16%), which was lower than 20% that described in other series [1, 20]. However, its association is also described in up to 30% of cases with other types of tumors, such as tumors of the ovary, endometrium, breast, kidney and liver [2, 4, 10]. Therefore, colorectal cancer should be ruled out in the postoperative period if a colonoscopy is not performed prior to surgery [17]. Similarly, careful examination of the ovaries is imperative at the time of surgery [4]. However, there is no established protocol on the most correct screening method.

Patient monitoring is recommended due to the risk of both associating other malignancies and progression of the same disease to PMP, even with an initial histology of benign neoplasia, as occurred in three of our cases.

## Conclusions

Mucinous appendiceal neoplasms can present with a variety of clinical manifestations. The condition may be an accidental finding in an imaging study for another cause or even during surgical intervention. The treatment is fundamentally surgical, although the technique depends on the histological findings.

Appendicular tumors require follow-up due to their potential malignant progression, even in benign neoplasms. In addition, given the high association they present with other neoplasms, it is necessary to screen tumor pathology at other levels.

## Data Availability

The dataset supporting the conclusions of this article is included within the article.

## Abbreviations

HIPEC: Hyperthermic intraperitoneal chemotherapy
PMP: Pseudomyxoma peritonei
